# First evidence of a new simian adenovirus clustering with *Human mastadenovirusF* viruses

**DOI:** 10.1186/s12985-019-1248-z

**Published:** 2019-11-27

**Authors:** Christian E. Lange, Fabien R. Niama, Kenneth Cameron, Sarah H. Olson, Rock Aime Nina, Alain Ondzie, Gerard Bounga, Brett R. Smith, Jasmine Pante, Patricia Reed, Ubald Tamufe, Anne Laudisoit, Tracey Goldstein, Romain Bagamboula MPassi, Damien O. Joly

**Affiliations:** 1 Metabiota Inc, Nanaimo, BC Canada; 2 National Laboratory of Public Health, Brazzaville, Republic of the Congo; 3 Wildlife Conversation Society, Bronx, NY USA; 4 Unites States Fish and Wildlife Service, Crossroads VA, Bailey’s, USA; 5 Ministry of Agriculture and Livestock, Brazzaville, Republic of the Congo; 6 One Health Institute, School of Veterinary Medicine, University of California, Davis, CA USA; 7 EcoHealth Alliance, New York, NY USA; 8 Ministry of National Defense, Brazzaville, Republic of the Congo; 9 British Columbia Ministry of Environment and Climate Change Strategy, Victoria, BC Canada

**Keywords:** Adenovirus, Primate, Zoonosis, Africa, Evolution, Bushmeat

## Abstract

**Background:**

Adenoviruses play an important role as human pathogens, though most infections are believed to be asymptomatic. The over 100 human adenovirus types are classified into seven species (A-G), some of which include simian adenoviruses. Recent findings have highlighted that simian adenoviruses have a zoonotic potential and that some human adenoviruses are likely the result of relatively recent spillover events.

**Methods:**

In order to evaluate the risks associated with primates hunted and sold as bushmeat, multiple samples from 24 freshly killed monkeys were collected in the Republic of the Congo and tested for adenovirus DNA by PCRs targeting the conserved DNA polymerase and hexon genes.

**Results:**

The DNA of a novel simian adenovirus was detected in a moustached monkey (*Cercopithecus cephus*) by the DNA polymerase PCR, but not by the hexon PCR. The 275 nucleotide amplicon was most closely related to members of the *Human mastadenovirus* *F* species (93% HAdV-40 and 89% HAdV-41 amino acid identity), rather than to other known simian adenoviruses.

**Conclusions:**

The phylogenetic clustering with *Human mastadenovirus* *F* sequences suggests a common ancestor, more recent than the last common ancestor of humans and moustached monkeys. The findings increase concerns about the zoonotic potential of simian adenoviruses and highlight the need for more research and surveillance on the issue.

## Background

Adenoviruses (AdVs) are double-stranded non-enveloped DNA viruses that infect a variety of vertebrate species including humans and primates. Adenoviruses were first discovered in the 1950s as human pathogens, and human adenoviruses (HAdVs) have been intensely studied since [1, 2]. Although the majority of adenovirus infections are largely asymptomatic, some HAdVs are responsible for a significant number of cases of respiratory diseases especially in children. HAdVs can also cause conjunctivitis, acute hemorrhagic cystitis, meningoencephalitis, diarrhea, intussusception, celiac disease and myocarditis [1, 2]. Despite their role as pathogens, some HAdVs and simian adenoviruses (SAdVs) have been used or proposed as tools in vaccine delivery and gene therapy [3]. The application of SAdVs has especially been explored in this context as a way to avoid potential pre-existing immune responses due to prior exposure to HAdVs.

The *Adenoviridae* family as a whole is quite diverse, with its currently five genera, *Mastadenovirus, Aviadenovirus, Atadenovirus, Siadenovirus* and *Ichtadenovirus*. The family is considered to be rather old, with virus host co-evolution playing a major role in its diversification [4]. Adenovirus types are named according to the host they were first isolated from, such as human, simian or bovine adenoviruses. All of the more than 100 described HAdV types fall within the *Mastadenovirus* genus, and they have been allocated to seven distinct species (*Human mastadenovirus A-G*) [http://hadvwg.gmu.edu]. Almost as many SAdVs have been described and allocated or proposed to belong to at least eight *simian mastadenovirus* species [5]. The distinction (nomenclature) between *human* and *simian adenovirus* species currently follows following principle: If an adenovirus species with primate hosts contains at least one HAdV type than the species is called a *human adenovirus* species, if not it is a *simian adenovirus* species. As a result, *Human mastadenovirus* species *B, C, E* and *G* include both, HAdVs and SAdVs [6].

The interaction between humans and their closest living relatives creates an inherent risk of pathogen transfer, which is an important concern for both, public health and wildlife conservation. The list of human pathogenic viruses that have been shown to be or are suspected as originating in primates is long and includes, for example, members of the retrovirus (HIV), filovirus (EBOV), herpesvirus (CeHV), and parvovirus (HBoV) families [7–10]. Although adenoviruses are generally considered to be rather host species specific viruses, there are some exceptions, such as canine adenovirus 1, which has a wider host range and can infect members for the *Canidae*, *Mustelidae* and *Ursidae* families [11]. The host range and zoonotic potential of simian adenoviruses has more recently become an area of interest, with at least three good examples underlining the relevance of this topic. First, the sequence analysis of HAdV-4 not only revealed a very close relationship with simian adenoviruses found in chimpanzees, but identified the likely origin of the virus to be a zoonotic event that created a recombination of an HAdV and an SAdV [3, 4, 12]. Second, the evaluation of HAdV-76, a virus found in a human patient but almost identical to SAdV-35 strains infecting chimpanzees and bonobos, revealed evidence of a back-and-forth transmission of this virus between humans and great apes [13]. Third, a spillover event of an adenovirus from titi monkeys into a human, followed by human-to-human transmission highlighted that such zoonotic events are not merely historic, but might have the potential to occur at any time [14]. Serological findings suggestive of exposure of laboratory personal following an adenovirus outbreak in a baboon colony also point in the same direction [15].

Hence, the goal of the study was to test primates hunted and sold as bushmeat for consumption in the Republic of the Congo (ROC) for adenovirus DNA in order to evaluate the risk of interspecies transmission of adenoviruses.

## Materials and methods

Samples were opportunistically collected from 24 freshly killed animals voluntarily provided by local hunters upon their return to the village following hunting trips. Hunters were not compensated to avoid an increase in hunting above normal rates. Samples of heart, lung, liver, kidney, and spleen were collected using sterile instruments and placed in individual 2.0 mL screw-top cryotubes containing 1.5 mL of RNAlater®, then stored at ambient temperature for 48 h prior to being frozen at − 80 °C. Samplers wore N95 masks, nitrile gloves, and protective eyewear during carcass sampling to minimize risks of disease transmission and sample contamination. DNA was manually extracted using Trizol® and was stored at − 20 °C until analysis. Two conventional consensus PCR assays, one nested, the other semi-nested, were used to test the samples for adenovirus DNA [16, 17]. The nested PCR targets the conserved DNA polymerase gene (~ 3500 nt) and yields a product of approximately 270-276 nt after primer trimming [16]. The semi-nested PCR targets the conserved DNA hexon gene (~ 2700 nt) and yields a product of approximately 334 nt between the primer binding sites. The primers of the latter PCR were specifically designed for the detection of a broad range of HAdVs [17].

PCR products were examined by gel electrophoresis on a 1.5% agarose gel and products of the expected amplicon sizes were excised, cloned, and the sequence determined by Sanger sequencing at the UC Davis DNA sequencing facility. The workflow within the laboratory was optimized to minimize the risks of contamination between different stages of the analysis process. Obtained sequences were analyzed in the Geneious software (version 7.1), and manually edited. Consensus sequences were compared to the GenBank database (BLAST N, NCBI). Since liver and lung tissue samples were collected and tested by both assays, an individual was considered positive for an adenovirus infection if adenovirus DNA was detected in either sample by either PCR method.

A maximum likelihood phylogenetic tree was constructed with the novel nucleotide sequence and sequences from the corresponding PCR target region of 46 other human and simian adenovirus sequences and two *Atadenovirus* sequences (*Bovine adenovirus 4* and *Ovine adenovirus 7*) as outgroup. Multiple sequence alignments were made in Geneious (version 11.1.3, MUSCLE Alignment), and regions supported by less than 50% of the sequences were excluded. Bayesian phylogeny of the polymerase gene fragment was inferred using MrBayes (version 3.2) with the following parameters: Datatype = DNA, Nucmodel = 4by4, Nst = 1, Coavion = No, # States = 4, Rates = Equal, 2 runs, 4 chains of 10,000,000 generations. The sequences of the two *Atadenoviruses* served as outgroup to root the trees, and trees were sampled after every 1000 steps during the process to evaluate phylogenetic convergence. The average standard deviation of split frequencies was below 0.0059 (MrBayes recommended final average < 0.01). The first 10% of the trees was discarded and the remaining ones combined using TreeAnnotator (version 2.5.1; http://beast.bio.ed.ac.uk) and displayed with FIGTREE (1.4.4; http://tree.bio.ed.ac.uk/).

## Results

**Fig. 1:**
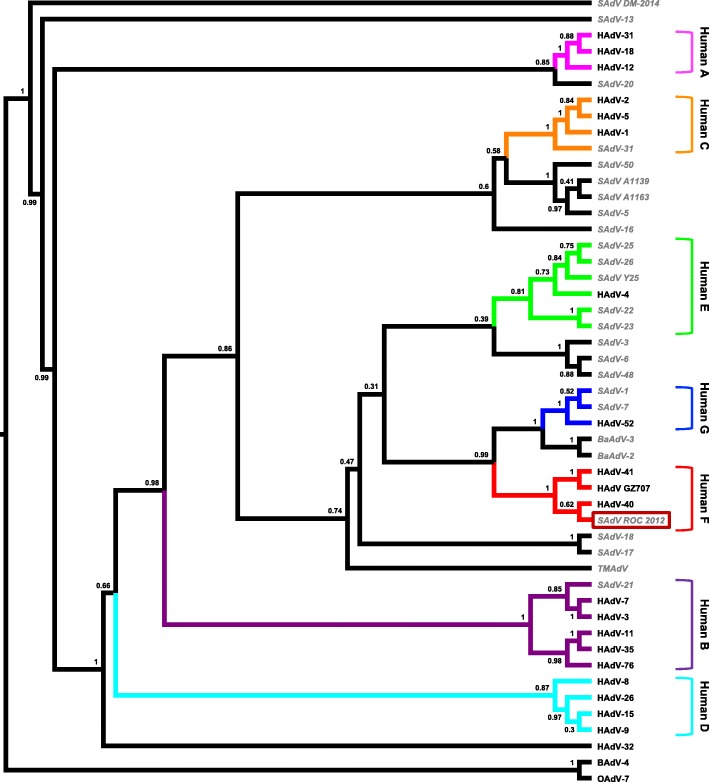
Phylogenetic tree of human and simian adenoviruses based on the PCR target region within the conserved Polymerase gene [15]. Numbers at the knods indicate the bootstrap support. The branches of the seven *Human mastadenovirus* species are differentiated by colors, *simian mastadenoviruses* grey and in italics for easier distinction. Sequences included are *Human mastadenovirus* HAdV-1 (AF534906), HAdV-2 (J01917), HAdV-3 (DQ086466), HAdV-4 (AY487947), HAdV-5 (M73260), HAdV-7 (AY495969), HAdV-8 (AB448767), HAdV-9 (AJ854486), HAdV-11 (AY163756), HAdV-12 (NC_001460), HAdV-15 (AB562586), HAdV-18 (GU191019), HAdV-26 (EF153474), HAdV-31 (AM749299), HAdV-32 (KF268327), HAdV-35 (AY271307), HAdV-40 (KU162869), HAdV-41 (DQ315364), HAdV-52 (DQ923122), HAdV GZ707 (KT369258) and HAdV-76 (KF633445), *simian mastadenoviruses* SAdV-1 (AY771780), SAdV-3 (AY598782), SAdV-5 (KP853111), SAdV-6 (CQ982401), SAdV-7 (DQ792570), SAdV-13 (KP329563), SAdV-16 (KP329564), SAdV-17 (KP329566), SAdV-18 (FJ025931), SAdV-20 (HQ605912), SAdV-21 (AR101858), SAdV-22 (AY539876), SAdV-23 (AY530877), SAdV-25 (AC000011), SAdV-26 (FJ025923), SAdV-31 (FJ025904), SAdV-48 (HQ241818), SAdV-50 (HQ241820), SAdV A1139 (JN880448), SAdV A1163 (JN880449), SAdV Y25 (JN254802) SAdV DM-2014 (KM190146), BaAdV-2 (NC_021168), BaAdV-3 (KC693023) and TMAdV (Titi monkey adenovirus ECC-2011; HQ913600), and bovine adenovirus 4 (AF036092) and ovine adenovirus 7 (NC_004037) from the *Atadenovirus* genus

Samples from 24 freshly hunted old world monkeys were collected between May and August 2012 at an established small village of approximately 30 individuals in the Sangha department in northern ROC. Hunted animals were either consumed by the hunters’ families, shared with other village households, or sold to passing vendors for transport to large regional markets. Most of the animals were guenons, represented by the species *Cercopithecus cephus* (9), *Cercopithecus neglectus* (1), *Cercopithecus nictitans* (10) and *Cercopithecus pogonias* (1), while three animals were mangabeys (*Lophocebus albigena*). Seven of the monkeys were female (4 adults, 3 juveniles) and 17 were male (12 adults, 5 juveniles) (Additional file 1). Adenovirus DNA was detected in the liver of one of the 24 animals (~ 4%), while none was amplified from any of the lung samples. The adenovirus DNA positive animal was a moustached monkey (*Cercopithecus cephus*). DNA was successfully amplified by the PCR targeting the DNA polymerase gene, but not by the PCR targeting the Hexon gene. The sequence corresponded to known adenovirus sequences, but was distinct from previously described ones. The closest matches for the newly isolated sequence in GenBank on the DNA level (BLASTN) were HAdV-40 (KU1628669) and HAdV-41 (MH465394) with 88% and SAdV-17 (KP329566) with 81% nucleotide identities at a 99% or higher query coverage. On the protein level the same viruses showed the highest amino acid identities with the isolate; 93% in case of HAdV-40, 89% in case of HAdV-41, and 86% in case of SAdV-17. Upon phylogenetic analysis the sequence clustered with HAdV-F species viruses, such as HAdV-40 and HAdV-41 (Fig. 1).

## Discussion

Direct human-primate interactions pose some zoonotic risks. Among potential spillover interfaces, hunting and consumption of primates as bushmeat poses particularly high risk because of the direct human contact with animal meat and bodily fluids [18]. Since adenoviruses are diverse and relatively common, there could be abundant exposure at this interface. The prevalence of adenovirus DNA we found in the studied animals (4%) was close to what was previously reported for liver samples (7%) and lung samples (1%) from primates [19]. This result confirms that hunters and consumers of bushmeat are exposed to SAdVs, but leaves open the question of whether these specific SAdVs can actually infect humans. Considering co-evolution of virus and host, phylogenetically closer hosts have a higher risk of pathogen cross transmission [20]. Since the majority of primates hunted and sold for consumption in Africa are not great apes, but rather smaller old world monkey species, this would be of lesser concern. Indeed the HAdVs where a relatively recent spillover between humans and primates occurred, seem to have originated from chimpanzees and bonobos [4, 12, 13].

Interestingly, the virus whose DNA we detected in a moustached monkey (*Cercopithecus cephus*) does not seem to be very closely related to other known SAdVs, but rather to human adenovirus F species viruses, such as HAdV-40 and HAdV-41. These two human viruses are known to circulate in ROC and are commonly involved in pediatric gastroenteritis; with a prevalence of 1–8% in respective patients in sub-Saharan Africa [21–23]. The conclusion of a close relationship between our isolate and HAdVs 40 and 41 is based on only a fragment of the DNA Polymerase gene, however a comparison of the PCR target region to the entire coding sequence among the viruses included in the phylogenetic analysis revealed, that the PCR product on average very accurately represents the entire sequence of this conserved gene. No DNA was amplified with the PCR targeting the hexon gene, despite a good sequence match between the primers and HAdVs 40 and 41. This could indicate, that the novel isolate and these viruses may differ more in the hexon gene. However, since the primers also match the hexon genomic sequences of the known SAdVs equally well, we rather suspect sensitivity issues with the PCR being the reason for its failure to amplify DNA from the sample. Considering the classification criteria, the new isolate would likely fall into the human adenovirus F species, and be the first SAdV in this species. The relatively close relationship with HAdVs implies a higher chance for the detected virus to be able to infect humans, and it seems likely that the new simian isolate and HAdV-40 and HAdV-41 have a common ancestor that is much younger than the last common ancestor of humans and moustached monkeys.

With regards to adenovirus evolution, these and previous findings allow for some interesting speculation. Based on the existing sequence data, human adenovirus species A, B, C, and D include primarily, and many, HAdV types. That probably indicates a long evolutionary history with the human host, as viruses in those species had the time to diversify from a common species ancestor. The presence of few SAdV types in the A-D HAdV species supports that hypothesis as their hosts are great apes and they are clearly distinct from the HAdVs in the species. In the human adenovirus species E, F and G however, we only know of one or two HAdVs each, and species E and G are dominated by SAdVs [24]. Considering the HAdV-4 data, the high sequence similarity of HAdV-52 with SAdVs and the findings presented here, it may be hypothesized that the known HAdVs in species E, F, and G did not originally co-evolve with humans, but rather are the product of more recent spillover events and consecutive adaptation. Since our knowledge about adenovirus diversity in primates, and even humans, is certainly still far from comprehensive, testing this hypothesis could provide new valuable insights into viral evolution and spillover risks.

## Conclusions

We conclude that our results, together with the previous reports about a recent origin of some HAdVs in great apes and the incidence of a SAdV transmission from titi monkeys directly to humans, followed by human-to-human transmission, suggest a high risk of cross-species transmission of adenoviruses between humans and primates. The risks associated with contact from wild, non-ape primates are likely underestimated, and additional surveillance and research is needed to better quantify such exposure.

## Supplementary information

**Additional file 1.** Excel file with details about the animals sampled for this study.

## Data Availability

The novel adenovirus sequence was deposited in GenBank under submission number MN136539.

## Abbreviations

AdV: Adenovirus
CeHV: Cercopithecine herpesvirus
DNA: Deoxyribonucleic acid
EBOV: Ebolavirus
HAdV: Human adenovirus
HBoV: Human bocavirus
HIV: Human immunodeficiency virus
nt: Nucleotide
PCR: Polymerase chain reaction
ROC: Republic of the Congo
SAdV: Simian adenovirus
